# Correlation analysis of the triglyceride glucose index and heart failure with preserved ejection fraction in essential hypertensive patients

**DOI:** 10.1002/clc.23881

**Published:** 2022-06-29

**Authors:** Li Ping Liao, Yang Yang, Yilin Wu, Weizhen Li

**Affiliations:** ^1^ Cardiology Department, Jiading Branch of Shanghai General Hospital Shanghai Jiao Tong University School of Medicine Shanghai China; ^2^ Cardiology Department The First Affiliated Hospital of Anhui Medical University Hefei Anhui China; ^3^ Cardiology Department, Shanghai General Hospital Shanghai JiaoTong University School of Medicine Shanghai China

**Keywords:** cardiac diastolic function, essential hypertension, heart failure with preserved ejection fraction, triglyceride glucose index

## Abstract

**Background:**

Triglyceride glucose index (TyG index) is a novel marker of insulin resistance. Studies have shown that TyG index is closely associated with the occurrence of hypertension and cardiovascular disease. However, little is known about the correlation between TyG index and the occurrence of heart failure with preserved ejection fraction (HFpEF) in hypertensive patients.

**Hypothesis:**

Our study assumes that TyG index strongly correlates with occurence of HFpEF in hypertensive patients.

**Methods:**

This research enrolled 559 hypertensive patients (273 patients with HFpEF and 286 without HFpEF) admitted to the Department of Cardiology of Jiading Branch of Shanghai General Hospital from 2020 to 2021 as the study subjects. Gender, age, diastolic blood pressure, systolic blood pressure (SBP), and heart rate (HR) were recorded at admission. Medication history and fasting blood samples were harvested after admission to detect laboratory index. Cardiac function and ventricular structure index were measured by echocardiography. Pearson correlation analysis was conducted to identify the correlation of TyG index with cardiac function and ventricular structure. The receiver operating characteristic (ROC) curve was used to evaluate the diagnostic value of TyG index in HFpEF with hypertension.

**Results:**

HFpEF patients had higher diuretic use frequencies, fasting plasma glucose, NT‐proBNP, triglycerides, TyG index, left atrial diameter (LAD), left ventricular mass index (LVMI), the ratio of peak E‐velocity of mitral orifice to peak velocity of early diastolic mitral annulus (E/e′), and SBP but lower ratio of peak E of early diastolic maximum blood flow velocity to peak A of late diastolic maximum blood flow velocity of mitral orifice (E/A) and average e′ than non‐HFpEF patients. Moreover, TyG index was correlated with LAD, left ventricular ejection fraction (LVEF), LVMI, average e′, E/e′, and NT‐proBNP. The multivariate regression analysis suggested that TyG index, E/e′, and NT‐proBNP were independent risk factors for HFpEF in hypertensive patients. Compared with E/e′ and NT‐proBNP, the area under the ROC curve (0.778 [95% confidence interval: 0.707–0.849]) was the largest for TyG index.

**Conclusion:**

TyG index is higher in HFpEF patients than in non‐HFpEF patients and related to cardiac diastolic function, which strongly correlates with occurrence of HFpEF in hypertensive patients.

## 1 INTRODUCTION

Heart failure, the final stage of cardiovascular disease, has been reported to have a higher mortality rate than any other cardiovascular disease.^1^ Patients with heart failure with preserved ejection fraction (HFpEF) account for approximately 50% of all patients with heart failure, with poor clinical prognosis.^2^ The clinical manifestations, biomarkers, and changes in cardiac structure and function are less diagnostic in HFpEF patients compared to patients with reduced ejection fraction.^3^ Therefore, the rate of misdiagnosis is high in HFpEF patients.

Studies have manifested that about 80% of HFpEF patients exhibit high blood pressure.^4^ Chronically elevated arterial blood pressure increases the pressure load of the left ventricle. The body compensates with the secretion of catecholamine and angiotensin II, which can cause myocardial cell hypertrophy and myocardial interstitial fibrosis to result in left ventricular hypertrophy and dilation.^5^ Also, elevated blood pressure is able to lead to a decrease in coronary blood flow reserve capacity and an increase in the risk of myocardial ischemia and atrial fibrillation, which are critical risk factors for the occurrence of HFpEF.^6^ Therefore, early diagnosis of HFpEF and prompt treatment are vital for hypertensive patients. It has been confirmed that insulin resistance (IR), metabolic disorders, and obesity assume a crucial role in the pathogenesis of heart failure.^7^ The homeostasis model assessment‐IR (HOMA‐IR) is the most widely accepted model for evaluation of IR. The triglyceride glucose (TyG) index is a surrogate indicator for IR^8^ and was verified by Calcaterra et al.^9^ to be highly correlated with HOMA‐IR, and the TyG index was more economical, simple, and widely applied.^9^ Zheng conducted a 9‐year longitudinal population‐based study, which enrolled 4686 subjects (3177 males and 1509 females), and the results displayed that TyG index was associated with the incidence of hypertension in Chinese.^10^ A recent study has demonstrated that TyG index is positively associated with type 2 diabetes and the incidence rate of cardiovascular diseases.^11^ It has also been documented that TyG index is related to poor prognoses in patients with heart failure and type 2 diabetes.^12^ However, little is known about the correlation between TyG index and the occurrence of HFpEF. This paper focuses on the observation of TyG index and HFpEF in hypertensive patients and further determines the relationship between TyG index and cardiac function.

## 2 RESEARCH SUBJECTS AND METHODS

### 2.1 Research subjects

The Haitai software (version 3.0) of the hospital was adopted to obtain clinical data of patients from the electronic medical record system. A total of 559 hypertensive patients admitted to the inpatient ward of the Department of Cardiology of Jiading Branch of Shanghai General Hospital from 2020 to 2021 were selected as the research subjects, including 291 males and 268 females. Their average age was 70.76 ± 12.13 years old.

### 2.2 Diagnostic criteria

Hypertension was diagnosed based on the standard definition of the 2020 International Society of Hypertension Global Hypertension Practice Guide.^13^ The blood pressure was measured repeatedly on different days, with the systolic blood pressure (SBP) in the consulting room of ≥140 mmHg and/or diastolic blood pressure (DBP) of ≥90 mmHg. HFpEF was diagnosed as per the diagnostic criteria for HFpEF in the 2016 European Society of Cardiology guidelines.^14^

Inclusion criteria are as follows: (1) signs or symptoms of heart failure; (2) elevated B‐type natriuretic peptide (BNP) levels (BNP > 35 pg/ml or NT‐proBNP >125 pg/ml); (3) evidence of cardiac structural alterations (the left atrial volume index ≥34 ml/m^2^ or the left ventricular mass index [LVMI] ≥ 115 g/m^2^ for males and 95 g/m^2^ for females) or functional features of diastolic dysfunction (ratio of early diastolic mitral flow velocity to early diastolic mitral ring velocity [E/e′] ≥13 and a mean e′ septal and lateral wall <9 cm/s]; (4) normal or slightly abnormal left ventricular ejection fraction (LVEF) ≥50% and not enlarged left ventricle;

Exclusion criteria are as follows: (1) white coat hypertension, defined as elevated blood pressure in the clinic despite mean ambulatory BP monitoring or consistent home BP readings <135/85 mmHg; (2) secondary hypertension (renal parenchymal hypertension, renovascular hypertension, primary aldosteronism, pheochromocytoma, increased cortisol, and coarctation of aorta); (3) congenital heart disease (atrial septal defect, ventricular septal defect, patent ductus arteriosus, patent foramen ovale, congenital pulmonary valve stenosis, congenital bicuspid aortic valve, ebstein anomaly, congenital aorticainus aneurysm, coronary artery fistulae, congenital tetralogy of Fallot); (4) severe heart valve disease (mitral stenosis, mitral regurgitation, aortic stenosis, and aortic incompetence); (5) restrictive cardiomyopathy; (6) hypertrophic cardiomyopathy; (7) pericardial diseases (acute pericarditis and constrictive pericarditis); (8) heart failure due to noncardiogenic factors (severe infection, anemia, and diseases of blood system); (9) thyroid dysfunction (hyperthyroidism and hypothyroidism); (10) severe liver damage (glutamic‐propylene and glutamic‐oxalacetic transaminases higher than three times the upper limit of normal); (11) heavy renal impairment (glomerular filtration rate <60 ml min^−1^ [1.73 m^2^]^−1^); (12) drugs affecting blood glucose and blood lipids used before admission.

### 2.3 Research methods

Gender, age, vital signs at admission (systolic blood pressure, diastolic blood pressure, and heart rate), medication history (calcium channel blocker [CCB]; beta‐blocker; angiotensin‐converting enzyme inhibitor/angiotensin receptor blocker [ACEI/ARB]; diuretics), and fasting blood samples were collected after admission to measure laboratory indicators: N‐terminal B‐type brain natriuretic peptide precursor (NT‐proBNP), uric acid, creatinine, alanine aminotransferase (ALT), aspartate aminotransferase (AST), high‐density lipoprotein cholesterol (HDL‐C), low‐density lipoprotein cholesterol (LDL‐C), total cholesterol (TC), triglyceride (TG), and fasting blood glucose (FBG).

### 2.4 Calculation of TyG index

TyG index is calculated with the following formula: TyG index = Ln (serum TG [mg/dl] × FBG [mg/dl]/2).

### 2.5 Indicators of cardiac echocardiography

All patients underwent routine echocardiography examination after admission. The following indicators were observed and recorded for all patients: left ventricular ejection fraction (LVEF), left ventricular end‐diastolic diameter (LVEDD), interventricular septal thickness (IVST), left ventricular posterior wall thickness (LVPWT), left atrial diameter (LAD), septal peak early diastolic mitral annulus velocity (septal e′), lateral wall early diastolic mitral annulus velocity (lateral wall e′), average e′ = (interval e′ + sidewall e′), the ratio of peak E‐velocity of mitral orifice to peak velocity of early diastolic mitral annulus (E/e′), and the ratio of peak E of early diastolic maximum blood flow velocity to peak A of late diastolic maximum blood flow velocity of mitral orifice (E/A). Left ventricular mass (LVM) was calculated according to the Devereux formula: LVM (g) = 1.04 × ([IVST + LVPWT + LVEDD]^3 ^− LVEDD^3^) − 13.6; LVMI (g/m^2^) = LVM/body surface product (BSA; m^2^), among which BSA (male) = 0.0057 × height (m) + 0.0121 × body weight (kg) + 0.0882 and BSA (female) = 0.0073 × height (m) + 0.0127 × body weight (kg) − 0.2106.

### 2.6 Statistical analysis

SPSS 26.0 software was employed for statistical analysis. All measurement data were first tested for normality, and the data conforming to normal distribution was represented by (*x* ± *s*). The *t*‐test was utilized for comparisons between the two groups. Data without normal distribution were expressed as M (P25, P75), and the nonparametric test was applied for comparisons between the two groups. Count data were displayed as an example (%), and the rank‐sum test was adopted for comparisons between the two groups. Pearson or Spearman correlation analysis was selected depending on whether the data were normally distributed. Logistic regression was used to analyze the relationship between HFpEF and various variables in patients with hypertension. The diagnostic efficiency of TyG index for HFpEF in hypertensive patients was evaluated using the receiver operating characteristic (ROC) curve. All tests were performed using the two‐sided test, with the test level set as *p* < .05.

## 3 RESULTS

### 3.1 Comparison of laboratory index between the HFpEF group and the non‐HFpEF group

There was no statistically significant difference between the HFpEF group and the non‐HFpEF group in terms of age, male, body mass index, atrial fibrillation, smoking, DBP, previous medication history (CCB, β‐blocker, and ACEI/ARB), the liver and kidney function, HDL‐C, and LDL‐C. A statistically significant difference was observed for SBP, the percentage of diuretics used, NT‐proBNP, FBG, TG, and TyG index between these two groups (*p* < .05) (Table 1).

**Table 1:** Comparison of clinical and laboratory baseline characteristics between HFpEF group and non‐HFpEF group

Variable	HFpEF (*n* = 286)	Non‐HFpEF (*n* = 273)	*p* value
Age (years)	52.26 ± 9.66	53.28 ± 9.64	.998
Male	155 (54.20)	150 (54.95)	.972
Body mass index (kg/m^2^)	24.28 ± 13.86	24.02 ± 13.53	.806
Atrial fibrillation	68 (23.68)	65 (23.81)	1.046
Smoking	141 (49.30)	169 (61.90)	.067
SBP, mmHg	143.79 ± 26.64	135.86 ± 19.19	.012
DBP, mmHg	81.51 ± 12.98	83.78 ± 15.73	.176
Previous medication history			
CCB	132 (46.15)	120 (43.96)	.705
β‐blocker	123 (43.01)	103 (37.73)	.483
ACEI/ARB	124 (43.71)	104 (38.10)	.466
Diuretics	90 (31.47)	30 (10.99)	<.001
Biochemical indicators			
FBG (mmol/L)	7.96 ± 2.98	6.37 ± 2.74	.033
NT‐proBNP (M[P_25_,P_75_]pg/ml)	588.16 (417.31, 774.56)	99.62 (87.70, 221.68)	<.001
Uric acid (μmol/L)	356.23 ± 89.18	348.51 ± 78.64	.520
Creatinine (μmol/L)	73.76 ± 15.04	71.69 ± 15.68	.314
ALT (U/L)	21.12 ± 10.46	22.36 ± 8.83	.445
AST (U/L)	23.07 ± 10.34	24.35 ± 13.66	.381
TG[M(P_25_,P_75_), mmol/l]	2.04 (1.63, 2.88)	1.48 (1.08, 1.86)	<.001
HDL‐C (mmol/L)	1.20 ± 0.38	1.28 ± 0.35	.104
LDL‐C (mmol/L)	2.38 ± 0.77	2.52 ± 0.85	.213
TC (mmol/L)	4.36 ± 0.92	4.31 ± 1.05	.301
TyG index	9.18 ± 1.08	6.66 ± 0.92	<.001

### 3.2 Comparison of echocardiographic indexes between the HFpEF group and the non‐HFpEF group

LAD, E/e′, and LVMI were higher but E/A and average e′ were lower in the HFpEF group than in the non‐HFpEF group (*p* < .05) (Table 2).

**Table 2:** Comparison of echocardiographic index between HFpEF group and non‐HFpEF group

Variable	HFpEF (*n* = 286)	Non‐HFpEF (*n* = 273)	*p* value
LVEDD (mm)	51.73 ± 10.03	50.45 ± 9.83	.343
LAD (mm)	47.28 ± 5.07	40.64 ± 4.71	.001
IVS (mm)	10.29 ± 2.08	9.93 ± 1.46	.172
LVEF (%)	0.64 ± 0.09	0.65 ± 0.07	.290
LVPWT(mm)	9.32 ± 1.26	9.16 ± 1.41	.363
LVMI (M[P_25_,P_75_],g/m^2^]	96.73 (77.46, 114.34)	81.14 (70.56, 90.98)	<.001
E/A	0.71 ± 1.03	1.31 ± 0.73	.003
Average e′ (cm/s)	5.94 ± 1.82	6.92 ± 1.62	<.001
E/e′ (M[P_25_,P_75_])	14.52 (11.06, 16.15)	8.68 (7.38, 10.40)	<.001

Abbreviations: HFpEF, heart failure with preserved ejection fraction; IVS, interventricular septal; LAD, left atrial diameter; LVEDD, left ventricular end‐diastolic diameter; LVEF, left ventricular ejection fraction; LVMI, left ventricular mass index; LVPWT, left ventricular posterior wall thickness.

### 3.3 Correlation analysis of TyG index with NT‐proBNP and echocardiographic indexes

The Spearman correlation analysis depicted that TyG index shared positive correlations with LVMI, LAD, E/e′, and NT‐proBNP and negative correlations with LVEF and average e′ (*p* < .05) (Table 3).

**Table 3:** Correlation analysis of TyG index between NT‐proBNP and echocardiographic indexes

Variable	*R* value	*p* value
LVEDD	0.053	.422
LAD	0.312	.001
IVS	−0.036	.698
LVEF	−0.468	.001
LVPWT	0.166	.083
LVMI	0.253	.002
E/A	−0.124	.203
Average e′	−0.205	.004
E/e′	0.266	.002
NT‐proBNP	0.406	<.001

Abbreviations: IVS, interventricular septal; LAD, left atrial diameter; LVEDD, left ventricular end‐diastolic diameter; LVEF, left ventricular ejection fraction; LVMI, left ventricular mass index; LVPWT, left ventricular posterior wall thickness; TyG, triglyceride glucose.

### 3.4 Multivariate step‐up logistic regression analysis of HFpEF in hypertensive patients

Variables with statistical differences in the univariate analysis were included in the logistics regression analysis with the occurrence of HFpEF as the dependent variable (yes = 1 and no = 0). The independent variables were assigned to diuretic or not (yes = 1 and no = 0), and the remaining continuous numerical variables were substituted with actual values. The analysis results exhibited that LVEF, TyG index, E/e′, and NT‐proBNP were influential factors for HFpEF (*p* < .05). Therefore, TyG index, E/e′, and NT‐proBNP were independent risk factors for HFpEF in patients with hypertension (Table 4).

**Table 4:** Risk factors of HFpEF analyzed by logistic regression analysis

Variable	Monofactor analysis			Multivariate analysis		
	OR	95% Cl	*p* Value	OR	95% Cl	*p* Value
LVEF	1.634	1.052–2.537	0.035	1.042	0.743–1.461	0.783
E/e′	1.716	1.105–2.664	0.028	1.252	1.010–1.623	0.003
TyG index	1.827	1.180–2.831	0.016	2.924	1.945–4.395	＜0.001
NT‐ProBNP	1.982	1.288–3.046	0.011	1.373	1.121–1.697	0.005

Abbreviations: CI, confidence interval; HFpEF, heart failure with preserved ejection fraction; LVEF, left ventricular ejection fraction; OR, odds ratio; TyG, triglyceride glucose.

### 3.5 ROC curve of TyG index, E/e,′ and NT‐proBNP to predict HFpEF in hypertensive patients

**Figure 1:**
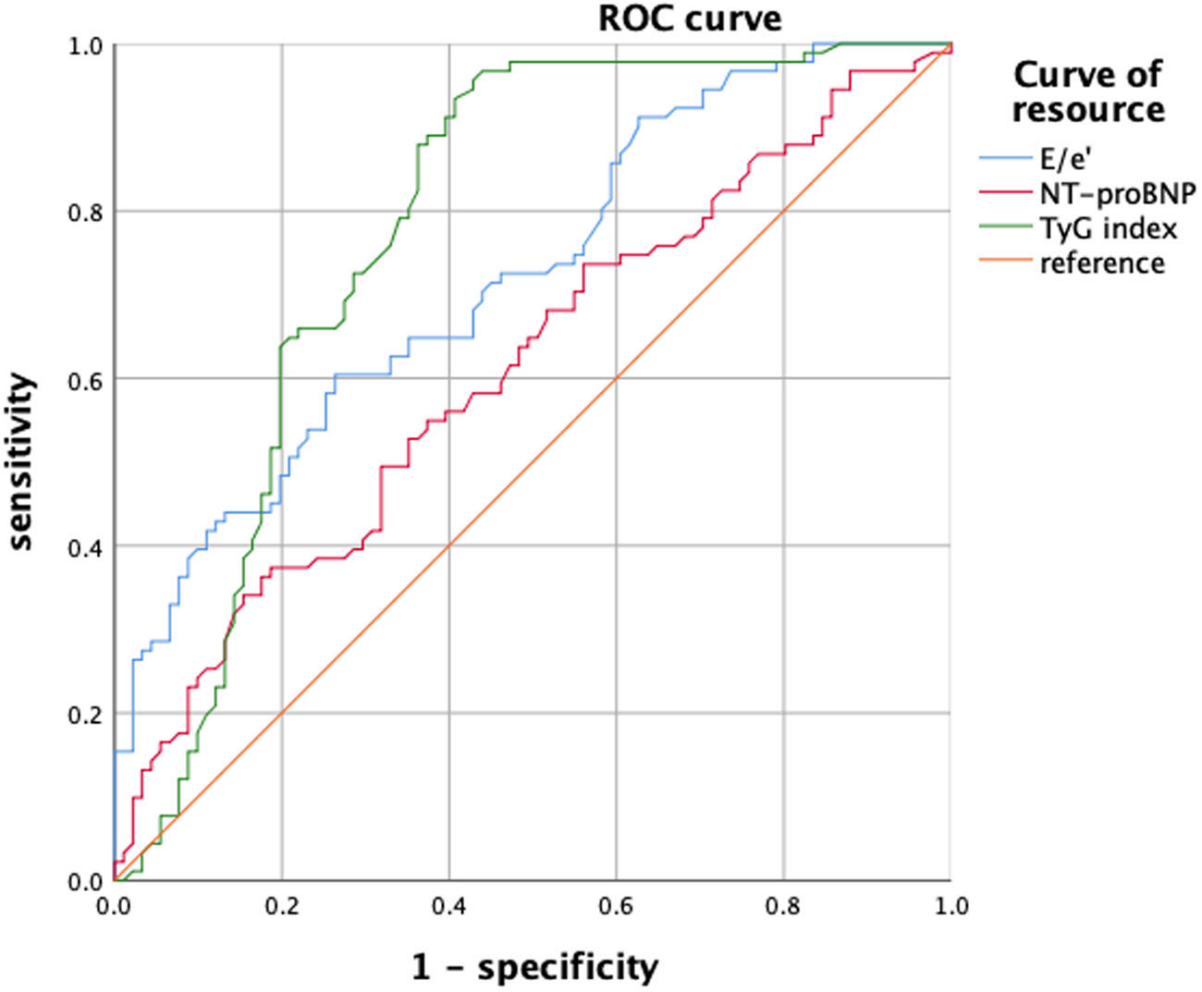
ROC curve of TyG index, E/e′, and NT‐proBNP to predict HFpEF in hypertensive patients. HFpEF, heart failure with preserved ejection fraction; ROC, receiver operating characteristic; TyG, triglyceride glucose.

The maximum area under the ROC curve to predict the occurrence of HFpEF in hypertensive patients was 0.722 (95% confidence interval [CI]: 0.649–0.794] for E/e′, 0.611 (95% CI: 0.530–0.693) for NT‐proBNP, and 0.778 (95% CI: 0.707–0.849) for TyG index (Figure 1).

## 4 DISCUSSION

This study delves into the relationship between TyG index and the occurrence of HFpEF in hypertensive patients. To our knowledge, this was the first study to explore the relationship between TyG index and HFpEF occurrence in patients with hypertension. It was noted that TyG index was significantly higher in HFpEF patients than in non‐HFpEF patients. High TyG index might be closely related to the incidence of HFpEF in hypertensive patients. The SBP in the HFpEF group was higher than that in the non‐HFpEF group. An earlier study also suggested that elevated blood pressure, especially SBP, could trigger decreased myocardial compliance, increased left ventricular filling pressure, and enlarged left atrium and pulmonary vein congestion, thus easy to lead to heart failure.^15^ Our data confirmed that NT‐ProBNP is higher in the HFpEF group, suggesting there's been activation of stretch receptors. NT‐ProBNP is recognized as a biomarker for assessing cardiac function and the severity of heart failure.^16^ TyG index was validated to be related to NT‐proBNP in our correlation analysis. The results were consistent with the conclusion obtained by the previous research of Guo et al.,^17^ which elucidated that the higher TyG index, the greater risk of chronic heart failure, illustrating worse cardiac function in patients. After admission, all patients underwent routine cardiac ultrasound to evaluate cardiac function. The results demonstrated that the HFpEF group showed higher LAD, LVMI, and E/e′ than the non‐HFpEF group, accompanied by lower E/A and average e′. All of these results indicated that hypertensive patients with HFpEF had poor cardiac structure and diastolic function.

TyG index was calculated using the formula of Ln (serum TG [mg/dl] × FBG [mg/dl]/2). It was first proposed by Guerreo Romero as a surrogate indicator of IR.^18^ The study of Alizargar et al. unveiled that TyG index was correlated with carotid atherosclerosis in hypertensive patients.^19^ Previous studies have elaborated that TyG index is associated with stroke,^20^ hyperuricemia,^21^ and coronary artery calcification^22^ in elderly patients with hypertension. A growing body of studies have confirmed that TyG index is related to the occurrence of cardiovascular and cerebrovascular diseases in patients with hypertension. In our study, logistic regression analyses indicated that after age, sex, and baseline features were adjusted, E/e′, NT‐proBNP, and TyG index were independent risk factors for the incidence of HFpEF in patients with hypertension. Negi et al. observed that NT‐proBNP, as a precursor of BNP, could reflect the function of left ventricular contraction and remodeling and has an important diagnostic value for HFpEF.^23^ Previous research has also unraveled that the increase of E/e′ was linked to the occurrence of heart failure. Based on the existing studies, it has been suggested that a possible mechanism is the association of E/e′ with the process of myocardial fibrosis.^24^ The area under the ROC curve of TyG index was higher than that of E/e′ and NT‐proBNP, indicating that TyG index had a better ability to predict HFpEF in patients with hypertension than E/e′ and NT proBNP.

The mechanism of TyG index in heart failure is not clear. According to the existing research, TyG index is considered to be related to IR. So far as we know, ventricular fibrosis has been proven to be one of the key intermediate links in the pathogenesis of heart failure.^25^ IR has been manifested to be associated with ventricular fibrosis. IR contributes to the deposition of lipids inside and outside cardiomyocytes, accelerating the process of ventricular fibrosis. Second, the renin‐angiotensin‐aldosterone system can be activated during IR to promote the reabsorption of sodium and water, which facilitates ventricular remodeling and leads to the occurrence of heart failure.^26^ Third, the ability of the myocardium to utilize free fatty acids and glucose is correspondingly weakened during IR, which easily causes a series of metabolic disorders in the body and reduces the tolerance of the myocardium to ischemia.^27^ Last but not the least, IR activates the sympathetic nervous system, enhances cardiac oxygen consumption, and aggravates myocardial injury.^28^

TyG index is a simple and economic indicator of IR, which is useful to help clinicians early screen out the “high‐risk group” prone to HFpEF in hypertensive patients and can be routinely monitored during the management of patients. It is recommended to add TyG index to the routine evaluation model of hypertensive population.

## 5 CONCLUSION

Our study shows that the TyG index strongly correlates with the occurrence of HFpEF in hypertensive patients.

## 6 LIMITATIONS

There exists several limitations in this study. First, this study is a single‐center retrospective study with a small sample size, selection bias, and no long‐term follow‐up of patients. In the future, multicenter studies with larger sample sizes are warranted to further confirm our conclusions. In addition, this study can only infer from our data that TyG may be implicated in the occurrence of HFpEF in hypertensive patients. The mechanism has not been clarified through relevant experiments. Finally, research is merited to further analyze the relationship between TyG index and the prognosis of heart failure in hypertensive patients with heart failure.

## CONFLICT OF INTEREST

The author declares no conflict of interest.

## Data Availability

Patients' data are protected by hospital ethics committees, and the data that support the findings of this study are available from the unanimous consent of hospital ethics committees and corresponding author upon reasonable request.
